# Immunotherapy for small cell lung cancer: current challenges and prospects

**DOI:** 10.1186/s40164-025-00720-w

**Published:** 2025-11-05

**Authors:** Jiaxin Zhong, Guangling Jie, Haorui Qin, Hongrui Li, Nuo chen, Patiguli Aerxiding, Xia Zou, Xiaomin Niu

**Affiliations:** 1 Department of Shanghai Lung Cancer Center, Shanghai Chest Hospital, Shanghai Jiao Tong University School of Medicine, 241 Huaihai West Road, Xuhui District, Shanghai, 200030 PR China; 2 Shanghai Key Laboratory of Thoracic Tumor Biotherapy, 241 Huaihai West Road, Xuhui District, Shanghai, 200030 PR China; 3 Key Laboratory of Systems Biomedicine (Ministry of Education), Shanghai Center for Systems Biomedicine, Center for Chemical Glycobiology, Zhang Jiang Institute for Advanced Study, Shanghai Jiao Tong University, Shanghai, 200240 China; 4 The Third Clinical Medical College of Xinjiang Medical University, Tumor Hospital Affiliated to Xinjiang Medical University, Urumgi, 830011 Xinjiang China

**Keywords:** Small cell lung cancer (SCLC), Immune checkpoint inhibitors (ICIs), Tumor–immune microenvironment (TIME), Antibody‒drug conjugates (ADCs)

## Abstract

Small cell lung cancer (SCLC) is a highly aggressive neuroendocrine malignancy with limited treatment options. While immunotherapy has revolutionized SCLC therapy and now serves as a front-line treatment, the disease continues to present significant clinical challenges. Despite therapeutic advances in lung cancer over the past decade, most SCLC tumors eventually reoccur. Growing insights into the tumor–immune microenvironment (TIME) highlights its critical role in SCLC progression and therapeutic resistance. In this review, we summarize distinctive features of the TIME in SCLC and evaluate current immunotherapeutic strategies, notably immune checkpoint inhibitors (ICIs), that have demonstrated survival benefits in a subset of patients. Furthermore, we explore emerging immunotherapeutic approaches and novel targets in SCLC, emphasizing the challenges limiting the successful application of immunotherapy in this disease.

**Supplementary Information:**

The online version contains supplementary material available at 10.1186/s40164-025-00720-w.

## Background

Small cell lung cancer (SCLC), a highly aggressive neuroendocrine malignancy, is characterized by rapid proliferation and early metastasis [1]. Owing to its aggressive nature, a considerable proportion of patients are diagnosed after the cancer has already spread [2–4]. SCLC accounts for approximately 15% of all lung cancers [5] and is strongly associated with smoking [6, 7]. On the basis of the extent of disease, SCLC is classified as limited-stage (LS-SCLC) or extensive-stage (ES-SCLC) [8]. While most patients exhibit an initial response to first-line therapy, relapse is almost inevitable and often occurs within one year [9]. Consequently, 5-year survival rates remain low (approximately 30% for LS-SCLC and under 10% for ES-SCLC) [7].

Recent progress in understanding the tumor-immune microenvironment (TIME) has facilitated the start of a new era of cancer immunotherapy. Immune checkpoint inhibitors (ICIs), such as cytotoxic T lymphocyte-associated protein 4 (CTLA-4) inhibitors and antibodies targeting programmed cell death protein 1 (PD-1) or its ligand PD-L1, have revolutionized the treatment of NSCLC [10]. In SCLC, however, immunotherapy has only recently been integrated into standard regimens. Since 2019, the combination of etoposide and platinum-based chemotherapy (EP) with ICIs has shown promise in improving survival outcomes [11]. The humanized monoclonal antibodies atezolizumab and durvalumab, both of which target PD-L1, have been approved in several countries for the first-line treatment of SCLC [12, 13].

Advances in genomic profiling and genetically engineered mouse models have yielded deeper insights into SCLC metastasis and plasticity, offering new avenues for therapeutic intervention [2, 14]. In this review, we summarize the defining characteristics of the TIME in SCLC, critically evaluate current immunotherapeutic approaches, and discuss the challenges and opportunities in advancing the application of immunotherapy for this aggressive malignancy. We also explore emerging strategies and novel therapeutic targets, aiming to provide insights that may ultimately improve survival outcomes and quality of life for patients with this devastating disease.

## The role of the immune microenvironment in SCLC

**Fig. 1:**
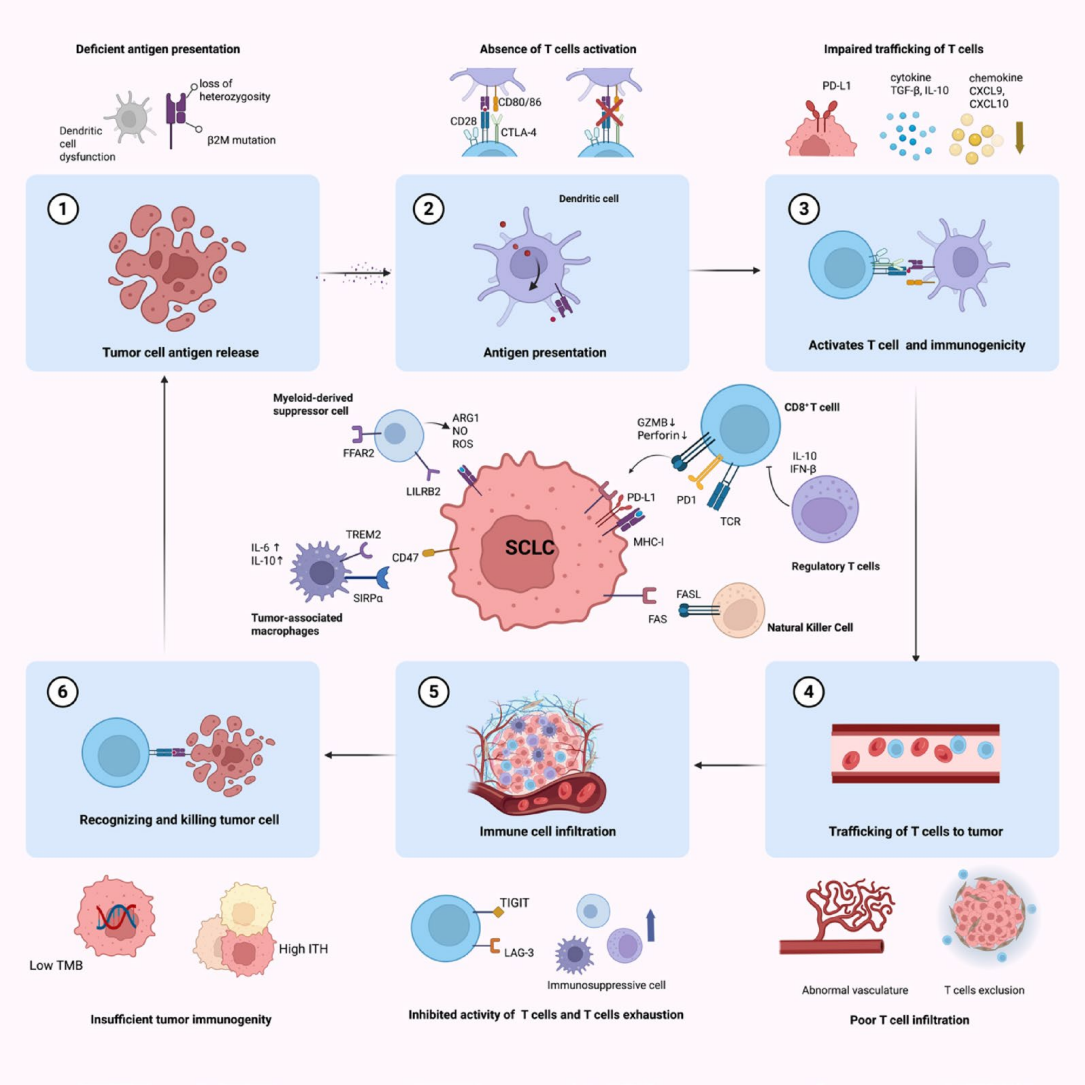
The cancer–immunity cycle and resistance mechanisms at each step, along with the functional landscape of immune cells within TIME of SCLC.The cancer–immunity cycle consists of six sequential steps (1.Tumor cell antigen release, 2. Antigen presentation, 3. Activates T cell and immunogenicity, 4. Trafficking of T cells to tumor, 5. lmmune cell infiltration, 6. Recognizing and killing tumor cell) and culminating in the recognition and elimination of cancer cells by immune effector cells. Various resistance mechanisms that impede each stage of this cycle are illustrated outside the loop, emphasizing how these barriers collectively contribute to the attenuated efficacy of ICIs. The central panel illustrates TIME of SCLC, highlighting the diverse immune cell populations and their respective tumor-promoting or tumor-suppressive functions. Created with biorender. com. Abbreviations: TMB, tumor mutational burden; ITH, intratumor heterogeneity; β2M, β2-microglobulin; CTLA-4, cytotoxic T-lymphocyte-associated protein 4; TGF-β, transforming growth factor beta; IL-6, interleukin-6; IL-10, interleukin-10; PD-1/PD-L1, programmed cell death protein 1 or its ligand; TCR, T-cell receptor; MHC-I, major histocompatibility complex class I; IFN-β, interferon-beta; CXCL9, CXC motif chemokine ligand 9; CXCL10, CXC motif chemokine ligand 10; TIGIT, T-cell immunoreceptor with immunoglobulin and ITIM domain; LAG-3, lymphocyte-activation gene 3; LILRB2, leukocyte immunoglobulin-like receptor B2; SIRPα, signal regulatory protein alpha; TREM2, triggering receptor xxpressed on myeloid Cells 2; FFAR2, free fatty acid Receptor 2; GZMB, granzyme B; ARG1, arginase-1; NO, nitric oxide; ROS, reactive oxygen species. FASL, FAS ligand

The tumor immune microenvironment (TIME) is a dynamic system composed of immune cells, stromal fibroblasts, the extracellular matrix (ECM), cytokines, and other factors [8]. Tumor cells interact, coexist, and compete with infiltrating immune cells and surrounding tissue, creating a unique microenvironment (Fig. 1) [15]. Owing to tumor heterogeneity and variations in immune system responses, the TIME is highly variable across individuals and evolves in response to tumor behavior [15, 16]. TIME exerts dual and often opposing influences: certain immune subsets suppress tumor growth, while others facilitate tumor progression and immune escape [17]. Understanding this delicate balance is critical for identifying why SCLC remains largely refractory to immunotherapy.

CD8⁺ T cells, also known as cytotoxic T lymphocytes (CTLs), play a pivotal role in tumor elimination. They recognize cancer cells through T-cell receptor (TCR) binding to major histocompatibility complex class I (MHC-I), and upon activation, induce cell death via FASL-mediated apoptosis or the release of perforin and granzymes [18]. Compared with lung adenocarcinoma (LUAD) and lung squamous cell carcinoma (LUSC), SCLC exhibits a markedly lower infiltration of CD8⁺ T cells [18], which may partly stem from reduced expression of the chemokine CCL5—a critical mediator of immune cell recruitment to the tumor microenvironment [19, 20]. Beyond quantitative depletion, SCLC-infiltrating CD8⁺ T cells often exhibit functional exhaustion characterized by elevated PD-1 expression, thereby attenuating cytotoxic activity and promoting immune evasion [21]. In addition, frequent loss or downregulation of MHC-I expression in SCLC further undermines antigen presentation, thereby impairing CD8⁺ T cells function [22].

Tumor-associated macrophages (TAMs) also play a critical role in tumor progression. Macrophages can adopt different functional states: M1 macrophages attack tumor cells, while M2 macrophages support tumor growth. The presence of M2-like macrophages is inversely correlated with survival in SCLC patients, suggesting that promoting the M2-to-M1 phenotype transition may be a potential therapeutic approach [23]. For example, macrophages in SCLC secrete interleukin-6 (IL-6), which activates the signal transducer and activator of transcription 3 (STAT3) signaling pathway to promote tumor growth [24]. Furthermore, the level of the chemokine CCL2, a potent recruiter of macrophages, is typically reduced in SCLC because of EZH2-mediated H3K27 methylation and DNMT1-mediated DNA methylation [25]. Another approach to enhance macrophage function is to target CD47. Blockade of CD47 can prevent it from binding to SIRPα, thereby inducing macrophage-mediated phagocytosis and enhancing antitumor immunity in SCLC [26]. In preclinical models of SCLC, the combination of radiotherapy with CD47 blockade activates macrophages and significantly improves antitumor efficacy [27].

As key components of the innate immune system, natural killer (NK) cells are essential for immune surveillance. Despite the high number of NK cells in the lungs [28], NK cell activity is impaired in the context of lung cancer, including SCLC [29]. Studies have shown that NK cell depletion can reduce the efficacy of combination therapies, such as the HDAC6 inhibitor ricolinostat (ACY-1215) in conjunction with JQ1 (bromodomain and extra terminal (BET) inhibitor), in treating SCLC [30]. Therefore, promoting NK cell infiltration in SCLC may enhance treatment responses. Some approaches explored in NSCLC include targeting the triggering receptor expressed on myeloid cells 2 (TREM2) receptor or activating the cGAS-STING pathway in tumor cells, both of which lead to increased NK cell infiltration [31, 32]. However, whether similar tactics are effective against SCLC remains to be determined.

Regulatory T cells (Tregs), a subset of T cells with immunosuppressive properties, inhibit T-cell activation and proliferation through contact-dependent mechanisms or the secretion of cytokines, such as IL-10 and IFN-β [33]. Patients with SCLC, especially those with ES-SCLC, often have increased proportions of Tregs, which can decrease the activity of CD8^+^ T cells [34, 35]. Additionally, TAMs in the SCLC microenvironment secrete IL-10, which can further promote Treg development by activating the FOXP3 pathway [23]. Given the role of Tregs in immune suppression, targeting FOXP3 or other aspects of Treg function may serve as potential therapeutic strategies to increase antitumor immunity in SCLC.

 Myeloid-derived suppressor cells (MDSCs) are another key component of the immunosuppressive tumor microenvironment. MDSCs inhibit T-cell proliferation and activation through the production of arginase 1 (ARG1), reactive oxygen species (ROS), and nitric oxide (NO). They can also contribute to tumor angiogenesis [36]. Certain receptors and pathways help MDSCs and other myeloid cells suppress immunity. For example, leukocyte immunoglobulin-like receptor B2 (LILRB2) on myeloid cells sends inhibitory signals and can outcompete CD8^+^ T cells for binding to HLA-I molecules. Blocking LILRB2 has been shown to reduce the infiltration of MDSCs and Tregs and push myeloid cells toward a more proinflammatory state [37]. Similarly, free fatty acid receptor 2 (FFAR2) inhibition can suppress MDSCs recruitment and reverse the immunosuppressive phenotype of the microenvironment [38]. Studies in SCLC have shown that all-trans retinoic acid (ATRA) induces the differentiation of MDSCs, whereas gemcitabine reduces their generation in the spleen, suggesting that therapeutic targeting of MDSCs may enhance the efficacy of immunotherapy in SCLC [16].

In summary, immune cells within the SCLC microenvironment play multifaceted roles, either facilitating or counteracting tumor progression. For example, recent findings indicate that while STAT3 promotes tumor growth during the initial stages of SCLC, paradoxically, knocking out STAT3 can increase metastatic potential [24, 39]. This duality makes it challenging to find the right therapeutic balance. A deeper understanding of how SCLC suppresses the immune response and how to bolster the immune system’s ability to fight tumors is imperative for advancing immunotherapeutic approaches in SCLC.

## Current immunotherapeutic approaches in SCLC

### The combination of immunotherapy and chemotherapy

**Fig. 2:**
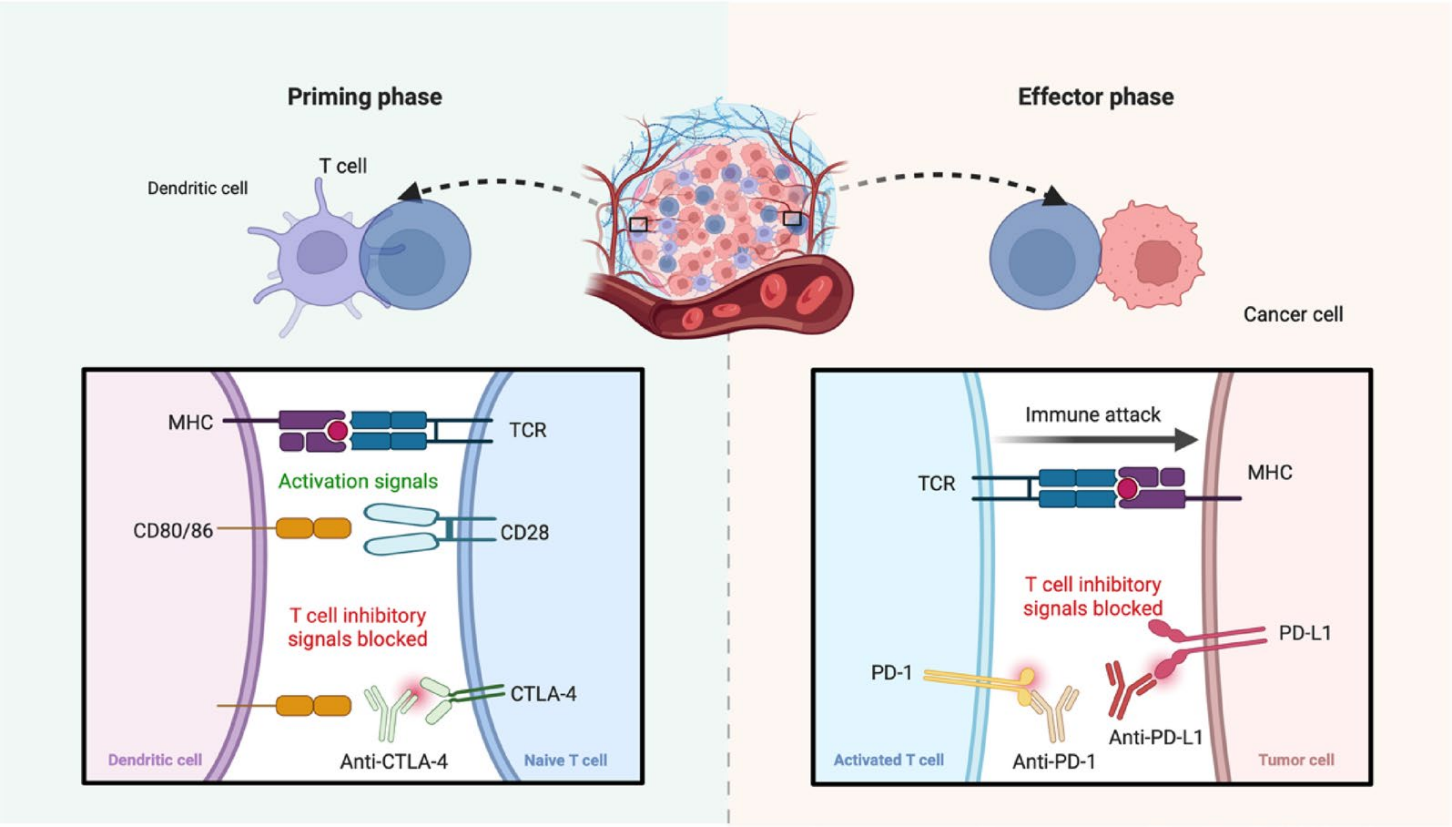
Mechanisms of action of CTLA-4 and PD-1/PD-L1. CTLA-4 primarily regulates T-cell activation during the priming phase in lymphoid organs by outcompeting the costimulatory receptor CD28 for binding to CD80/CD86 on dendritic cells, thereby inhibiting full T-cell activation. In contrast, PD-1 exerts its inhibitory effects primarily in peripheral tissues during the effector phase, where its interaction with PD-L1 on tumor cells or antigen presenting cells to suppresse T-cell activity, cytokine production, and cytotoxic function. Blocking the CTLA-4 or PD-1/PD-L1 pathway with ICIs can restore T-cell function and enhance antitumor immunity. Created with biorender.com

Platinum-based chemotherapy has long been the standard of care for SCLC. Although many patients initially respond to chemotherapy, these responses are not durable, and resistance commonly develops, leading to relapse. However, recent advancements in the understanding of the TIME and the development of ICIs have led to promising new therapeutic strategies [40]. PD-1/PD-L1 inhibitors are the main ICIs currently used in clinical practice (Fig. 2) [41].

The PD-1/PD-L1 axis plays a critical role in tumor immune evasion. PD-1, which is expressed on T cells, interacts with its ligand PD-L1, which is found on tumor cells and other components of the TIME, suppressing T-cell-mediated cytotoxicity and facilitating tumor survival and progression. Inhibiting this interaction with PD-1/PD-L1 can restore the antitumor immune response [42]. However, PD-1/PD-L1 inhibitor monotherapy has shown limited efficacy in SCLC compared with NSCLC, potentially due to the low presence of tumor-infiltrating lymphocytes (TILs) and the immunosuppressive phenotype of the SCLC microenvironment [43].

Recent clinical trials have provided hope for improved outcomes when PD-1/PD-L1 inhibitors are combined with chemotherapy (Table 1). In the IMpower133 study (NCT02763579), 403 patients with ES-SCLC were randomized to receive atezolizumab plus carboplatin-etoposide (*n* = 201) or placebo plus carboplatin-etoposide (*n* = 202). The addition of atezolizumab extended the median progression-free survival (PFS) from 4.3 months to 5.2 months (hazard ratio [HR], 0.77; 95% confidence interval [CI], 0.62–0.96; *P* = 0.020) and extended the median overall survival (OS) from approximately 10.3 months to 12.3 months (HR, 0.76; 95% CI, 0.60–0.95; *P* = 0.015). Additionally, atezolizumab achieved a high overall response rate (ORR) of 60%. Importantly, the incidence of grade ≥ 3 adverse events (AEs) was comparable between the atezolizumab group and the chemotherapy alone group (58.6% vs. 57.6%) [44]. Similarly, the CASPIAN study (NCT03043872) of 805 patients revealed that the addition of durvalumab to platinum-etoposide significantly improved the median OS compared with that with chemotherapy alone (12.9 months vs. 10.5 months; HR, 0.75; 95% CI, 0.62–0.91; *P* = 0.003) [45, 46]. In another phase III study, RATIONALE-312, the combination of the PD-1 inhibitor tislelizumab with chemotherapy, yielded a significant improvement in PFS (4.7 months vs. 4.3 months; HR, 0.64; 95% CI, 0.52–0.78; *P* < 0.0001). Tislelizumab treatment also led to a median OS of 15.5 months versus 13.5 months with chemotherapy alone (HR 0.75; 95% CI, 0.61–0.93; *P* = 0.004), echoing the benefit observed in IMpower133 [47]. Importantly, the incorporation of PD-1/PD-L1 inhibitors did not lead to an increased incidence of AEs, underscoring their favorable safety profile and promising therapeutic potential.

**Table 1:** Overview of current treatment regimens for SCLC

Study	Clinical phase	Brain metastasis at baseline	Number	Treatmemt	ORR	PFS (months)		OS (months)	Treatment-related AEs grade ≥ 3	The most common treatment-related AEs grade ≥ 3	
Immunotherapy											
IMpower133 (NCT02763579)	phaseIII	17 (9%)	201	Atezolizumab + carboplatin + etoposide	0.60	5.2 (HR = 0.77; *P* = 0.020)	12.3 (HR = 0.76; *P* = 0.015)		116 (59%)	Rash	40 (20%)
		18 (9%)	202	Placebo + carboplatin + etoposide	0.64	4.3	10.3		113 (58%)		21 (11%)
CASPIAN (NCT03043872)	phase III	28 (10%)	268	Durvalumab + platinum + etoposide	0.68	5.1 (HR = 0.80)	12.9 (HR = 0.75; *P* = 0.003)		171 (65%)	Neutropenia	64 (24%)
		38 (14%)	268	Durvalumab + tremelimumab + platinum + etoposide	0.58	4.9 (HR = 0.84)	10.4 (HR = 0.82; *P* = 0.045)		196 (73%)		85 (32%)
		27 (10%)	269	Platinum + etoposide	0.58	5.4	10.5		173 (65%)		88 (33%)
RATIONALE-312 (NCT04005716)	phase III	1 (< 1%)	227	Tislelizumab + etoposid + cisplatin/carboplatin	0.68	4.7 (HR = 0.64; *P* < 0.0001)	15.5 (HR = 0.75; *P* = 0.004)		194 (85%)	Neutropenia	127 (56%)
		4 (2%)	230	Placebo + etoposide + cisplatin/carboplatin	0.62	4.3	13.5		197 (86%)		125 (55%)
CAPSTONE-1 (NCT0371130)	phase III	5 (2%)	230	Adebrelimab + etoposide + carboplatin	0.71	5.8 (HR = 0.67; *P* < 0.0001)	15.3 (HR = 0.72; *P* = 0.002)		197 (86%)	Neutropenia	174 (76%)
		5 (2%)	232	Placebo + etoposide + carboplatin	0.66	5.6	12.8		197 (85%)		175 (75%)
ASTRUM-005 (NCT0406316)	phase III	50 (13%)	389	Serplulimab + etoposide + carboplatin	0.84	5.7 (HR = 0.48)	15.4 (HR = 0.63; *P* < 0.001)		129 (33%)	Neutropenia	55 (14%)
		28 (14%)	196	Placebo + etoposide + carboplatin	0.74	4.3	10.9		54 (28%)		27 (14%)
EXTENTORCH (NCT04012606)	phase III	3 (1%)	223	Toripalimab + etoposide + cisplatin/carboplatin	0.78	5.8 (HR = 0.67; *P* < 0.001)	14.6 (HR = 0.80; *P* = 0.030)		199 (90%)	Neutropenia	165 (74%)
		4 (2%)	219	Placebo + etoposide + cisplatin/carboplatin	0.73	5.6	13.3		193 (89%)		162 (75%)
KEYNOTE-604 (NCT03066778)	phase III	33 (15%)	228	Pembrolizumab + etoposide + platinum	0.71	4.5 (HR = 0.75; *P* = 0.002)	10.8 (HR = 0.80; *P* = 0.016)		177 (79%)	Neutropenia	97 (44%)
		22 (10%)	225	Placebo + etoposide + platinum	0.62	4.3	9.7		170 (76%)		91 (41%)
Anti-angiogenesis											
ETER701 (NCT04234607)	phase III	25 (10%)	246	Benmelstobart + anlotinib + etoposide + carboplatin	0.81	6.9 (HR = 0.32; *P* < 0.0001)		19.3 (HR = 0.61; *P* = 0.0002)	229 (93%)	Neutropenia	171 (70%)
		22 (9%)	245	Anlotinib + placebo + etoposide + carboplatin	0.81	5.6 (HR = 0.44; *P* < 0.0001)		13.3 (HR = 0.86; *P* = 0.172)	230 (94%)		178 (73%)
		26 (10.5%)	247	Placebo + etoposide + carboplatin	0.67	4.2		11.9	214 (87%)		169 (69%)
BEAT-SC (jRCT2080224946)	phase III	38 (23%)	167	Atezolizumab + bevacizumab + etoposide + cisplatin/carboplatin	0.82	5.7 (HR = 0.70; *P* = 0.006)		13 (HR = 1.22; *P* = 0.221)	147 (86%)	/	/
		27 (16%)	166	Atezolizumab + placebo + etoposide + cisplatin/carboplatin	0.73	4.4		16.6	149 (91%)	/	/
ALTER1202 (NCT03059797)	phase II	/	67	Anlotinib	0.05	4.0 (HR = 0.19; *P* < 0.0001)		7.3 (HR = 0.46; *P* = 0.006)	24 (36%)	Hypertension	9 (13%)
		/	34	Placebo	0.03	0.7		4.4	7 (21%)	GGT elevation	3 (9%)
Radiotherapy											
ADRIATIC (NCT03703297)	phase III	/	264	Chemoradiotherapy + durvalumab	0.30	55.9 (HR = 0.73; *P* = 0.010)		16.6 (HR = 0.76; *P* = 0.020)	64 (24%)	Pneumonitis or radiation pneumonitis	8 (3%)
		/	266	Chemoradiotherapy + placebo	0.32	33.4		9.2	64 (24%)	Pneumonia	9 (3%)
MATCH (NCT04622228)	phase II	/	56	Low-dose radiotherapy + atezolizumab + etoposide + cisplatin/carboplatin	0.88	6.9		NR	/	Neutropenia	34 (61%)

Abbreviations: PFS, progression-free survival; OS, overall survival; ORR, objective response rate; AEs, adverse events; HR, hazard ratio; GGT, gamma-glutamyl transpeptidase; NR, not reported

The landmark IMpower133 trial paved the way for immunotherapy in SCLC, and subsequent studies have continued to validate its efficacy. In the ASTRUM-005 trial, compared with chemotherapy alone, the PD-1 inhibitor serplulimab plus etoposide/carboplatin (EC) chemotherapy improved OS by more than four months [48]. Similar positive outcomes have been reported in studies such as CAPSTONE-1 (NCT03711305) [49] and EXTENTORCH (NCT04012606) [50], which also support the efficacy of combining immunotherapy with chemotherapy as a first-line treatment for SCLC.

CTLA-4, a negative regulator of T-cell activation, is highly expressed in many tumors and is associated with a poor prognosis. It competes with CD28 for binding to the B7 family of costimulatory molecules, thereby inhibiting T-cell activation [51]. Blocking CTLA-4 can promote antitumor immunity; for example, CTLA-4 inhibitors have been shown to increase the function and number of cytokine-induced killer (CIK) cells and increase the production of IFN-γ, a key cytokine in antitumor immunity [52]. However, the results of clinical trials of CTLA-4 inhibitors in SCLC have been largely disappointing. In a CA184-156 trial (NCT01450761) with 1132 SCLC patients, treatment with ipilimumab did not significantly improve the median OS, and AEs were more frequent in the ipilimumab group [53, 54]. One possible explanation for this lack of efficacy is that while ipilimumab may stimulate peripheral T-cell activation, it may be less effective at activating T cells within the tumor microenvironment. Similarly, in a subgroup analysis of the CASPIAN study, the combination of durvalumab and tremelimumab with platinum-etoposide did not improve the median OS (10.4 months vs. 10.5 months; HR = 0.82; 95% CI, 0.68–1.00.68.00; *P* = 0.045) and was associated with a greater incidence of AEs, treatment-related termination, and death [55, 56].

Recently, an increasing number of clinical trials have explored the synergistic potential of combining immunotherapy with other modalities, such as surgery, radiotherapy, chemotherapy, and targeted therapies. These studies aimed to provide more tailored and effective treatment options for SCLC patients (Table 1).

### Anti-angiogenic therapy combined with immunotherapy and chemotherapy

Tumors often secrete factors such as vascular endothelial growth factor (VEGF) that promote new blood vessel formation (angiogenesis), which can also suppress the immune response. The combination of immunotherapy with antiangiogenic drugs is being investigated in SCLC. The ETER701 trial (NCT04234607) investigated the efficacy of a novel PD-L1 inhibitor, benmelstobart, combined with the antiangiogenic drug anlotinib and standard chemotherapy. Compared with the combination of anlotinib/EC, the combination of benmelstobart/anlotinib/EC significantly improved both PFS (6.9 months vs. 5.6 months) and OS (19.3 months vs. 13.3 months). These findings indicate that combining antiangiogenic therapy with immunochemotherapy holds promise as an effective treatment strategy for SCLC patients. However, the trial also highlighted a high rate of treatment interruption due to AEs associated with the four-drug combination, suggesting that the toxicity associated with the four-drug combination needs careful consideration and management [57]. Additionally, the phase III BEAT-SC trial (jRCT2080224946) is currently investigating the therapeutic potential of ICIs in combination with antiangiogenic agents, further exploring the role of this strategy in SCLC treatment. Separately, the ALTER1202 trial (NCT03059797) demonstrated that anlotinib alone exhibited notable efficacy in patients with relapsed SCLC, further supporting the therapeutic potential of targeting angiogenesis in this setting [58].

### Radiotherapy combined with immunotherapy and chemotherapy

Prophylactic cranial irradiation (PCI) has been shown to reduce the risk of brain metastases in SCLC patients, but its benefit in ES-SCLC patients is questionable and is being re-evaluated in the context of immunotherapy, especially given the potential for cognitive side effects [59, 60]. New approaches, such as hippocampal avoidance-prophylactic cranial irradiation (HA-PCI), have been shown to protect cognitive function without compromising efficacy, suggesting a way to make PCI safer [61]. Additionally, the ADRIATIC trial (NCT03703297) is being performed to assess a novel approach involving immunoconsolidation therapy following concurrent chemoradiotherapy for LS-SCLC, which may reshape second- and later-line treatment guidelines. In this study, patients who received durvalumab after concurrent chemoradiotherapy achieved significantly longer OS compared with those in the placebo group (55.9 months vs. 33.4 months; HR, 0.73; 98% CI, 0.54–0.98; *P* = 0.010). PFS was also improved with durvalumab (16.6 months vs. 9.2 months; HR, 0.76; 97% CI, 0.59–0.98; *P* = 0.020) [62].

Recent studies have shown that low-dose computed tomography (LDCT) for lung cancer screening can provide significant survival benefits. The National Lung Screening Trial (NLST), which involved 53,000 patients aged 55 to 74 years, demonstrated that compared with chest radiography, LDCT reduced lung cancer mortality by 20% and reduced overall mortality by 6.7% [63]. Early detection of SCLC through such screening may reveal patients at an earlier, more treatable stage, which may in turn improve the efficacy of subsequent therapies, including immunotherapy.

Building on the success of IMpower133, the MATCH trial (NCT04622228) sought to explore the combination of low-dose radiotherapy (LDRT; 15 Gy in 5 fractions) with immunotherapy. The combination yielded a median PFS of 6.9 months, and analysis of patient samples suggested that radiation may increase the proportion of stem-like CD8^+^ T cells associated with stronger antitumor activity. However, owing to the relatively small sample size of the study, further research is needed to validate these findings and assess the broader applicability of this combined approach [64].

## Emerging strategies and novel targets in SCLC immunotherapy

While most well-established ICIs target PD-1/PD-L1 and CTLA-4, several ICIs with novel therapeutic targets have emerged for SCLC, offering promising avenues for treatment (Table 2). In addition, we summarize the ongoing clinical trials investigating these novel ICIs to provide a comprehensive overview of current research progress (Table 3). The targets are discussed as follows.

**Table 2:** Emerging therapeutic strategies and novel targets in SCLC

Study	Clinical phase	Brain metastasis at baseline	Number	Treatmemt	ORR	PFS (months)	OS (months)	Treatment-related AEs grade ≥ 3	The most common treatment-related AEs grade ≥ 3	
DLL3										
TRINITY (NCT02674568)	phase II	134 (40%)	339	Rovalpituzumab tesirine	0.42	3.5	5.6	135 (40%)	Thrombocytopenia	37 (11%)
TAHOE (NCT03061812)	phase III	175 (59%)	296	Rovalpituzumab tesirine	0.15	3.0 (HR = 1.51)	6.3 (HR = 1.46)	183 (64%)	Dyspnea	22 (7%)
		87 (59%)	148	Topotecan	0.21	4.3	8.6	113 (88%)	Neutropenia	49 (38%)
MERU (NCT03033511)	phase III	54 (15%)	372	Rovalpituzumab tesirine	0.09	3.7 (HR = 0.51)	8.8 (HR = 1.12, *P* = 0.237)	217 (59%)	Thrombocytopenia	34 (9%)
		56 (16%)	376	Placebo	0.05	1.4	9.9	111 (30%)	Increased aspartate aminotransferase	6 (2%)
NCT03026166	phase I/II	21 (70%)	30	Rovalpituzumab tesirine + nivolumab	0.28	4.8	7.4	16 (53%)	Anemia, Pericardial effusion, Pneumonitis, Pleural effusion	3 (10%)
		8 (67%)	12	Rovalpituzumab tesirine + nivolumab + ipilimumab	0.36	4.1	11	11 (92%)	Thrombocytopenia	3 (25%)
DeLLphi-300 (NCT03319940)	phase I	38 (25%)	152	Tarlatamab (AMG 757)	0.25	3.5	17.5	150 (99%)	Cytokine release syndrome	50 (33%)
PARP										
NCT01638546	phase II	12 (22%)	55	Temozolomide + veliparib	0.39	3.8 (HR = 0.84; *P* = 0.390)	8.2 (*P* = 0.500)	/	Thrombocytopenia	27 (50%)
		10 (20%)	49	Temozolomide + placebo	0.14	2.0	7.0	/	Lymphopenia	12 (26%)
NCT02446704	phase I/II	20 (40%)	50	Olaparib + Temozolomide	0.42	4.2	8.5	/	Neutropenia	19 (38%)
ECOG-ACRIN 2511 (NCT01642251)	phase II	0	64	Veliparib + cisplatin + etoposide	0.72	6.1 (HR = 0.75; *P* = 0.060)	10.3 (HR = 0.83; *P* = 0.170)	/	Neutropenia	32 (49%)
		0	64	Placebo + cisplatin + etoposide	0.66	5.5	8.9	/	Neutropenia	21 (32%)
NCT02289690	phase II	/	61	Veliparib + chemotherapy followed by veliparib maintenance	0.77	5.8 (HR = 0.67; *P* = 0.059)	10.1 (HR = 1.43; *P* = 0.088)	49 (82%)	Neutropenia	34 (57%)
		/	59	Veliparib + chemotherapy followed by placebo	0.59	5.7 (HR = 0.98; *P* = 0.924)	10.0 (HR = 1.46; *P* = 0.083)	51 (88%)	Neutropenia	32 (55%)
		/	61	Placebo + chemotherapy followed by placebo	0.64	5.6	12.4	41 (68%)	Neutropenia	24 (40%)
S1929 (NCT04334941)	phase II	13 (24%)	54	Atezolizumab + talazoparib	0.11	2.9 (HR = 0.66; *P* = 0.019)	9.7 (HR = 0.98; *P* = 0.470)	30 (57%)	Anemia	19 (37%)
		10 (19%)	52	Atezolizumab	0.19	2.4	9.5	9 (18%)	AST increased	2 (4%)
TIGIT										
SKYSCRAPER-02 (NCT04256421)	phase III	47(19%)	243	Tiragolumab + atezolizumab + carboplatin + etoposide	0.71	5.1 (HR = 1.08)	12.8 (HR = 1.09; *P* = 0.421)	127 (53%)	Rash	63 (26%)
		46 (19%)	247	Placebo + atezolizumab + carboplatin + etoposide	0.66	5.4	12.9	142 (58%)	Rash	47 (19%)
B7-H3										
IDeate-Lung01	phase II	19 (41%)	46	I-DXd (DS-7300) 8 mg/kg	0.26	4.2	9.4	20 (44%)	Neutropenia	1 (2%)
		18 (43%)	42	I-DXd (DS-7300) 12 mg/kg	0.55	5.5	11.8	21 (50%)	Neutropenia	7 (17%)
Epigenetic therapies										
NCT03879798	phase I/II	/	21	Valemetostat (DS-3201b) + irinotecan	0.21	2.2	6.6	/	Anemia	6 (29%)
Other										
NCT02454972	phase II	4 (4%)	105	Lurbinectedin	0.35	3.5	9.3		Fatigue	7 (7%)
ATLANTIS (NCT02566993)	phase III	46 (15%)	307	Lurbinectedin + doxorubicin	0.32	4.0 (HR = 0.83)	8.6 (HR = 0.97; *P* = 0.900)	145 (48%)	Neutropenia	112 (37%)
		49 (16%)	306	Topotecan or cyclophosphamide, doxorubicin, and vincristine	0.29	4.0	7.6	218 (75%)	Neutropenia	200 (69%)
LUPER (NCT04358237)	phase I/II	6 (21%)	28	Lurbinectedin + pembrolizumab	0.46	4.6	10.5	23 (82%)	Neutropenia	19 (44%)
TROPiCS-03 (NCT03964727)	phase II	5 (12%)	43	Sacituzumab govitecan (SG)	0.42	4.4	13.6	26 (61%)	Neutropenia	13 (47%)

Abbreviations: AST, aspartate aminotransferase

**Table 3:** Ongoing clinical trials targeting novel therapeutic pathways in SCLC

Study	Population	Drug	Function	Phase	Line	Sample size	State	Primary end point
DLL3								
NCT04429087	SCLC; progressed 1 line of platinum-based treatment(including anti-PD-1/PD-L1)	Obrixtamig (BI 764532)	T-cell connectors targeting DLL3	phase I	≥ 2	/	Recruiting	MTD
DAREON-9 (NCT05990738)	SCLC; progressed 1 line of platinum-based treatment(including anti-PD-1/PD-L1)	Obrixtamig (BI 764532) + topotecan/single agent chemotherapy	T-cell connectors targeting DLL3	phase 1b	≥ 2	25	Recruiting	DLTs, MTD
NCT05978284	SCLC; progressed 1 line of platinum-based treatment(including anti-PD-1/PD-L1)	ZG006	DLL3/DLL3/CD3 trispecific T-cell engager	phase II	≥ 2	48	Recruiting	DLTs, MTD
NCT06592638	SCLC; progressed 1 line of platinum-based treatment(including anti-PD-1/PD-L1)	ZG006	DLL3/DLL3/CD3 trispecific T-cell engager	phase I	≥ 2	/	Recruiting	DLTs, MTD
IDE849-001 (NCT07174583)	SCLC; progressed 1 line of platinum-based treatment(including anti-PD-1/PD-L1)	IDE849	DLL3 ADC	phase I/II	≥ 2	/	Recruiting	Safety and Tolerability
DNA damage response modulator								
NCT05815160	SCLC; progressed 1 line of platinum-based treatment(including anti-PD-1/PD-L1)	Debio0123 + etoposide + carboplatin	WEE1 kinase inhibitors	phase I	≥ 2	16	Recruiting	DLTs
B7-H3								
NCT06954246	SCLC; progressed 1 line of platinum-based treatment(including anti-PD-1/PD-L1)	MHB088C	B7-H3 ADC	phase I/II	≥ 2	91	Recruiting	ORR, OS
IDEate-Lung01 (NCT05280470)	SCLC; progressed 1 line of platinum-based treatment(including anti-PD-1/PD-L1)	Ifinatamab Deruxtecan (I-DXd)	B7-H3 ADC	phase II	≥ 2	137	Active, not recruiting	ORR

Abbreviations: DLTs, dose limiting toxicities; MTD, maximum tolerated dose; ADC, antibody drug conjugate

### Delta-like ligand 3 (DLL3)

**Fig. 3:**
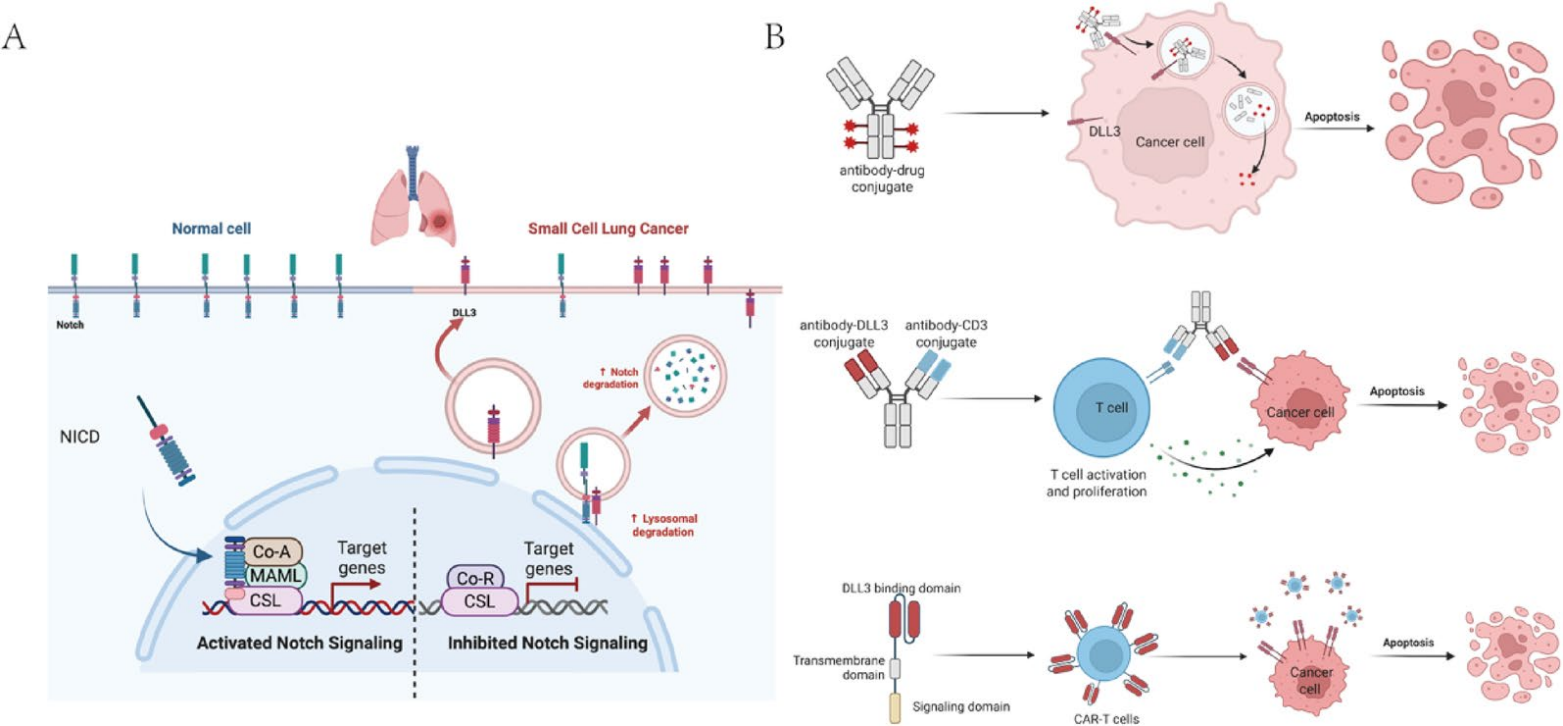
DLL3 overexpression and therapeutic targeting strategies in SCLC. **A** In SCLC, the overexpression of DLL3, an inhibitory ligand of the Notch signaling pathway, leads to the suppression of Notch activity, contributing to tumor progression. **B** Therapeutic strategies targeting DLL3 include antibody‒drug conjugates, bispecific T-cell engagers, and chimeric antigen receptor T-cell (CAR-T) therapies. Created with biorender. com. Abbreviations: NCID, Notch intracellular domain; DLL3, delta-like ligand 3; Co-A, coactivator; MAML, mastermind-like; CSL, CBF1/suppressor of hairless/Lag1, Co-R, corepressor. CAR-T, chimeric antigen receptor T cell

The Notch signaling pathway, a critical mediator of cell development and differentiation, is often dysregulated in various cancers. DLL3, a ligand of the Notch pathway, functions as a negative regulator, inhibiting Notch signaling (Fig. 3A) [65]. DLL3 is highly overexpressed in SCLC tumors compared to normal tissues. This selective expression has garnered considerable attention as a potential therapeutic target [65, 66]. Notably, DLL3 expression varies across SCLC molecular subtypes. In atypical SCLC (aSCLC)—a distinct subset characterized by the absence of *RB1* and *TP53* co-inactivation and by limited or no tobacco exposure—DLL3 expression closely parallels that of ASCL1 [67]. This co-expression pattern indicates that DLL3 is transcriptionally regulated by ASCL1, thereby establishing a mechanistic rationale for subtype-oriented precision therapies targeting the ASCL1–DLL3 signaling axis in SCLC.

Strategies to target DLL3 include antibody‒drug conjugates (ADCs), bispecific T-cell engagers (BiTEs), and chimeric antigen receptor T-cell (CAR-T) therapies (Fig. 3B) [68]. Rovalpitumab tesirine (Rova-T), an ADC targeting DLL3, was discontinued after phase II trials (TRINITY, NCT02674568) failed to demonstrate significant clinical efficacy and was associated with severe toxicities, including pleural effusion, decreased appetite, photosensitivity reactions, and dyspnea [69]. These adverse effects may be caused by premature drug release from Rova-T or the paracellular effects of drug cleavage, which may lead to off-target toxicity. In light of these setbacks, researchers have turned to alternative therapeutic approaches [70]. Efforts are underway to increase antigen recognition and improve the safety and efficacy of ADCs, including the development of immune-stimulating ADCs, conditionally active ADCs and protein-degrading ADCs, among other novel approaches [71]. Despite the setbacks in DLL3 ADC trials such as TAHOE (NCT03061812) and MERU (NCT03033511) [70, 72], recent studies have explored the combination of Rova-T with ICIs. In a trial (NCT03026166) that combined Rova-T with nivolumab, the median PFS was 4.8 months, and the median OS was 7.4 months. When Rova-T was combined with both nivolumab and ipilimumab, the median PFS was 4.1 months, whereas the median OS improved to 11.0 months. Although these results are promising, significant toxicity was reported, and many patients were unable to tolerate the combination [73]. Overall, there have been challenges in the application of DLL3 ADCs, prompting a shift toward alternative approaches.

One promising alternative strategy is the use of BiTEs, which bind DLL3 on tumor cells to CD3 on CTLs, thereby inducing T-cell activation, inflammatory cytokine release, and CTL-mediated tumor cell death [74]. This approach facilitates the targeted recruitment of T cells into tumor tissue, resulting in tumor regression [75]. Tarlatamab (AMG757), a first-generation BiTE, has shown better efficacy and a more favorable toxicity profile in early-phase clinical trials. The DeLLphi-300 study (NCT03319940), a phase I trial evaluating tarlatamab, demonstrated that tarlatamab produced higher response rates at higher doses. In the latest data update, among 152 patients who received doses of ≥ 10 mg, the median PFS and median OS were reported as 3.5 months and 17.5 months, respectively, with an ORR of 25%. The main side effect is cytokine release syndrome, which mostly occurs in the first treatment cycle, and it is generally manageable [66, 76]. Subsequent trials have revealed an optimal dosing schedule [77] and are exploring tarlatamab in combination with chemotherapy. Preliminary data indicate improved efficacy [78]. Given its encouraging clinical performance, tarlatamab has been approved for the treatment of patients with SCLC with disease progression following chemotherapy. Multiple clinical trials, including NCT04429087 [79] and DAREON-9 (NCT05990738), are ongoing to further assess the efficacy of DLL3-targeted BiTEs in SCLC.

In addition to BiTEs, CAR-T-cell therapies targeting DLL3 have demonstrated potent antitumor effects in preclinical studies [80, 81]. However, early indications are that DLL3-targeted BiTE combined with PD-L1 blockade may be superior to CAR-T cells combined with PD-L1 blockade [82]. Interestingly, engineering CAR-T cells to secrete interleukin-18 (IL-18) can improve their persistence and activity, helping them overcome the tumor immunosuppressive environment and facilitating tumor control [83]. Additionally, recent advancements in radioimmunotherapy targeting DLL3 indicate encouraging antitumor activity with minimal toxicity, highlighting its potential as an important future therapeutic modality for SCLC [84].

### Poly (ADP‒ribose) polymerase (PARP)

PARP is a key enzyme crucial for repairing damaged DNA [85]. PARP inhibitors, which prevent cancer cells from repairing DNA breaks, have been shown to be effective in cancers with DNA repair deficiencies (such as cancers with *BRCA1/2* mutations) [86]. Although BRCA mutations are rare in SCLC, a substantial proportion of SCLC patients exhibit clinical responses to PARP inhibitors. One theory for this sensitivity is that the degradation of lineage-specific oncoproteins may sensitize SCLC cells to DNA damage and PARP inhibition [87]. Additionally, PARP inhibitors have been shown to activate innate immune pathways, promoting T-cell infiltration and upregulating PD-L1 expression. In SCLC, combining PARP inhibition with radiotherapy elevates the levels of chemokines such as CCL5 and CXCL10, effectively turning an “immune desert” tumor into an “immune-enriched” tumor that is more receptive to PD-1/PD-L1 blockade [88–90]. These findings suggest that PARP inhibitors may not only kill tumor cells directly but may also make the tumor more visible to the immune system.

Several clinical trials have explored the use of PARP inhibitors in SCLC. In a phase II clinical trial (NCT01638546), adding the PARP inhibitor veliparib to temozolomide significantly increased the ORR (39% vs. 14%, *P* = 0.016), although it did not prolong PFS [91]. Another phase I/II clinical trial compared the combination of olaparib and temozolomide and demonstrated superior therapeutic effects to those of veliparib. This trial reported an ORR of 42% and a median PFS of 4.2 months, indicating that olaparib may offer enhanced clinical benefits in SCLC patients [92]. Notably, among six patients with aSCLC treated with temozolomide, four paitients (67%) achieved treatment durations exceeding 10 months. These observations indicate that temozolomide may confer more durable responses and clinical benefit in aSCLC compared with conventional SCLC. However, the limited sample size necessitates larger studies to validate efficacy and to clarify the predictive value of relevant biomarkers [67].

In the ECOG-ACRIN 2511 study (NCT01642251), patients were randomized 1:1 to receive either veliparib combined with cisplatin and etoposide or placebo with the same chemotherapy regimen. The median PFS in the veliparib arm was 6.1 months (HR = 0.75; *P* = 0.060), indicating a potential therapeutic signal, although the degree of improvement was limited. The OS was 10.3 months (HR = 0.83; *P* = 0.170), indicating no statistically significant benefit [93].

### T-cell immunoglobulin and the ITIM domain (TIGIT)

TIGIT is an immune checkpoint receptor that, like PD-1, can suppress the antitumor immune response. It engages with the ligand CD155, and through this interaction, TIGIT can increase the release of the immunosuppressive cytokine IL-10 and decrease the secretion of the proinflammatory cytokine IL-12 [94]. TIGIT also competes with an activating receptor (DNAM-1) for CD155 binding, thereby reducing the costimulatory signals essential for optimal immune activation. Furthermore, when TIGIT is engaged, it directly decreases NK cell activity through its inhibitory signaling motifs [95].

Because TIGIT is often expressed alongside PD-1 on antigen-specific T cells in tumors (including SCLC), it has become an attractive target for immunotherapy. One strategy involves the use of bispecific antibodies that block both TIGIT and PD-L1. This combinatorial approach has potential for enhancing immune-mediated tumor elimination in SCLC, and several clinical trials are currently investigating the efficacy of such dual-targeted therapies [96].

For example, the phase III SKYSCRAPER-02 trial (NCT04256421) evaluated the addition of the anti-TIGIT antibody tiragolumab to standard atezolizumab plus chemotherapy, although this particular study did not show an improvement in outcomes [97]. Despite these limitations, TIGIT remains a promising immunotherapeutic target, and combination approaches (such as TIGIT plus PD-1/PD-L1 blockade) continue to be explored.

### Epigenetic therapies

Unlike NSCLC, SCLC has few obvious druggable mutations, but it exhibits significant epigenetic dysregulation, such as abnormal DNA methylation and histone modification [98, 99]. While the potential of targeting these alterations has been recognized, the current landscape of FDA-approved epigenetic drugs is largely limited to hematological cancers, highlighting a critical gap in the treatment of solid tumors such as SCLC [100].

One notable epigenetic target is lysine-specific demethylase 1 (LSD1), an enzyme that regulates histone modification by demethylating histone H3 at lysine 4 (H3K4), which is expressed at high levels in SCLC. Inhibiting LSD1 can slow tumor cell growth and may enhance immune recognition by upregulating MHC-I on cancer cells [101, 102]. However, a clinical trial of an LSD1 inhibitor (GSK2879552) in SCLC had to be stopped owing to unfavorable side effects [103]. Another key epigenetic regulator is enhancer of zeste homolog 2 (EZH2), a histone methyltransferase that catalyzes the methylation of H3K27. EZH2 overactivation, driven by *RB1* mutations and subsequent E2F overexpression, leads to the silencing of tumor suppressor genes and the activation of oncogenic signaling pathways. EZH2-induced DNA methylation contributes to chemotherapy resistance and immune evasion in SCLC [104]. Furthermore, chromodomain Y-like (CDYL) may regulate DNA methylation by recruiting EZH2 enhancers, further exacerbating resistance [105]. Blocking EZH2 in combination with stimulating the STING immune pathway may reverse immune resistance in SCLC models by increasing T-cell activity and tumor recognition [106].

### Others

In addition to the emerging targets discussed above, several other therapeutic approaches are being investigated for the treatment of SCLC. Lymphocyte-activation gene 3 (LAG-3) is another inhibitory receptor on T cells. Antibodies blocking LAG-3 have been shown to promote antitumor immune response in head and neck squamous cell carcinoma (HNSCC). Multiple clinical trials are testing LAG-3 blockers alone or combined with other ICIs in SCLC, and early data suggest that they may become important components of future treatment strategies [95].

DExD/H-box helicase 9 (DHX9) is an RNA/DNA helicase involved in the regulation of RNA and DNA functions, as well as in the maintenance of genomic stability. Inhibiting DHX9 in cancer cells can increase DNA damage and stress, potentially facilitating the recognition of tumors by the immune system. Research indicates that targeting DHX9 in SCLC may increase immune cell infiltration and activation, thereby improving the response to ICIs [107].

A novel transcription factor, NFIC, has recently been implicated in the regulation of glucose metabolism in SCLC. Targeting NFIC presents an opportunity to disrupt the metabolic pathways that sustain tumor growth. One promising strategy involves the use of bromodomain-containing protein 4 (BRD4) inhibitors or bromodomain and extraterminal domain inhibitors (BETis) to downregulate NFIC expression, potentially inhibiting tumor progression and increasing the efficacy of existing treatments [108].

CAR-T cell therapies have shown remarkable success in treating hematologic malignancies, yet their application in solid tumors such as SCLC has been limited. Disialoganglioside GD2 (GD2), a glycosphingolipid expressed on the surface of most SCLC tumors, has emerged as a target for CAR-T cell therapy. Studies in both in vitro and in vivo SCLC models have demonstrated the potential of GD2-directed CAR-T cells to induce tumor regression [109]. Moreover, CAR-T cells derived from pluripotent stem cells (iPSCs) targeting GD2 exhibit enhanced cytotoxic activity, suggesting a novel approach for treating SCLC and addressing the challenges associated with solid tumor immunotherapy [110].

Lurbinectedin is an RNA polymerase II inhibitor that prevents transcription factors from binding to gene promoters, thereby inhibiting tumor transcription [111]. In a trial of relapsed SCLC, lurbinectedin as a second-line treatment achieved a median PFS of 3.5 months and a median OS of 9.3 months. Notably, patients with a chemotherapy-free interval of ≥ 90 days prior to treatment demonstrated significantly improved outcomes [112]. The ongoing phase III ATLANTIS trial (NCT02566993), which investigated the combination of lurbinectedin and doxorubicin in patients with relapsed SCLC, has also shown improvements in overall survival [113]. Another study, the LUPER trial (NCT04358237), assessed the efficacy of lurbinectedin in combination with pembrolizumab in patients with relapsed SCLC following chemotherapy. The results indicated that combination therapy was more effective in platinum-sensitive patients (chemotherapy-free interval ≥ 90 days) than in platinum-resistant patients (chemotherapy-free interval < 90 days). In the platinum-sensitive cohort, the median PFS and OS were 8.0 months and 15.7 months, respectively, whereas the median PFS and OS were notably shorter (2.8 months and 7.1 months) in the platinum-resistant cohort. These findings suggest that lurbinectedin-based combination therapy may be a promising approach for select SCLC patients, particularly those with longer chemotherapy-free intervals [114].

Trophoblast cell surface antigen 2 (Trop-2) is a tumor-associated calcium signaling protein closely linked to cancer proliferation. Sacituzumab govitecan, an ADC that targets Trop-2, exhibits high tumor selectivity and bioavailability while maintaining a favorable toxicity profile [115, 116]. This agent has demonstrated therapeutic efficacy across multiple solid tumors [117–119] and is now being further investigated in SCLC [120].

## Challenges in immunotherapy for SCLC

SCLC is an exceedingly aggressive malignancy, underscoring the urgent need to investigate the underlying mechanisms of drug resistance and immune evasion. Such research is crucial for the development of more precise and effective therapeutic strategies.

### Drug resistance

In recent years, epithelial‒mesenchymal transition (EMT)-mediated drug resistance has emerged as a major challenge in SCLC treatment. Specifically, claudin (CLDN) 1 can reduce the sensitivity to drugs by activating the TGF-β1/EMT signaling pathway. Experimental treatments such as EMT inhibitors (tranilast and zoledronic acid) have shown potential in reversing this resistance [121]. Other pathways implicated in resistance include the erythropoietin-producing hepatocellular A2 (EphA2) signaling network, which can lead to the methylation of SOX2, thereby promoting EMT and chemoresistance [122]. Interestingly, different molecular subtypes of SCLC show distinct resistance patterns: the SCLC-P subtype is associated with EMT, whereas the SCLC-I subtype is associated with endothelial-to-mesenchymal transition (EndMT). Certain drugs can target these processes. For example, the BET inhibitor JQ1 has shown sensitivity to EndMT-driven resistance [123]. Similarly, interference with other resistance factors, such as contactin 1 (CNTN-1), is being explored as a way to resensitize tumors to therapy [124]. Triptolide, which has shown potential in reversing EMT-induced resistance in lung adenocarcinoma, has yet to be evaluated in SCLC [125]. Researchers are also investigating targeted therapies that can block specific resistance pathways; for example, the MET inhibitor crizotinib can counteract HGF/MET-induced EMT and has shown potential in restoring chemosensitivity in preclinical studies [126].

Another major factor in SCLC chemoresistance is the loss of a protein called Schlafen 11 (SLFN11). SLFN11 plays a pivotal role in tumor replication and growth by regulating DNA replication at replication forks. In SCLC, SLFN11 expression is correlated with the efficacy of topoisomerase I/II inhibitors, alkylating agents, and PARP inhibitors [127]. Many chemoresistant SCLC tumors have low SLFN11 levels, often due to epigenetic silencing. The enzyme EZH2 can repress SLFN11, and there are ongoing trials testing EZH2 inhibitors to determine whether they can restore SLFN11 expression and overcome resistance. However, the initial results have been underwhelming, likely because of suboptimal drug concentrations and a short treatment duration [128, 129]. Nevertheless, SLFN11 also predicts the response to PARP inhibition. In a phase II trial (NCT02289690), patients whose tumors expressed SLFN11 had longer progression-free survival when treated with a PARP inhibitor (veliparib) plus chemotherapy than did those whose tumors did not express SLFN11 (7.5 vs. 5.8 months, HR 0.6; 80% CI 0.36–0.97; *P* = 0.200) [130].

Another trial, the S1929 trial (NCT04334941), specifically enrolled SLFN11-positive patients and reported that adding the PARP inhibitor talazoparib to immunotherapy improved outcomes compared with immunotherapy alone (PFS 2.9 vs. 2.7 months, HR = 0.64; 80% C 0.49–0.85; *P* = 0.019) [131]. Researchers are also exploring ways to reactivate silenced SLFN11, using drugs such as HDAC inhibitors to reverse SLFN11 gene methylation or combining other drugs (Ataxia telangiectasia mutated and Rad3-related (ATR) inhibitors) with agents such as lurbinectedin to attenuate resistance in SLFN11-low tumors [132, 133]. Given the limited availability of tumor biopsies in advanced SCLC, SLFN11 expression in circulating tumor cells (CTCs) is a noninvasive biomarker for monitoring treatment response and resistance, facilitating personalized therapeutic strategies [134].

In addition to SLFN11-related pathways, the DNA repair capabilities are often enhanced in SCLC tumors, enabling tumor cells to survive treatment with chemotherapy, which works by damaging DNA. For example, tumors with enhanced checkpoint kinase 1 (CHK1) activity or other DNA damage response (DDR) pathways can quickly overcome chemotherapy-induced damage [135]. In recent years, researchers have identified several drugs that target DDR pathways to sensitize tumors to chemotherapy. Notably, agents targeting CHK1 and PARP have shown potential in overcoming resistance in SCLC [136]. A novel concept in SCLC resistance is nucleophagy, in which tumor cells selectively degrade parts of their own nucleus to eliminate damaged DNA. The protein HMGB1, a protein involved in DNA repair and chromatin structure, can induce auto-PARylation of PARP and upregulate LC3, a protein associated with autophagy. This process promotes nucleophagy and helps to remove damaged nuclear components, further enhancing the ability of tumors to resist treatment-induced DNA damage [137].

Another key factor contributing to SCLC drug resistance is the expression of CD133, a stem cell marker that is associated with increased expression of BCL-2 and p-glycoprotein (P-gp), both of which are involved in resistance to chemotherapy [138]. BCL-2 in particular helps cancer cells avoid cell death, so drugs targeting BCL-2 are being explored in SCLC. Venetoclax, a BCL-2 inhibitor already used in hematologic malignancies, has shown activity in preclinical SCLC models with high BCL-2 expression. Treatment with venetoclax caused significant tumor cell death and even tumor regression in these models, and notably, SCLC cells did not quickly develop resistance to venetoclax in short-term experiments [139, 140]. Combining venetoclax with other therapies has yielded even stronger antitumor effects than venetoclax alone in laboratory studies, suggesting that BCL-2 inhibition may be a valuable addition to combination treatment strategies for SCLC [141].

### Immune evasion

The upregulation of PD-L1 is well recognized as a mechanism of immune escape in many solid tumors, as it binds to PD-1 receptors on T cells and inhibits their activation. However, in SCLC, the upregulation of PD-L1 is only observed in a subset of patients, suggesting that other immune evasion mechanisms play a more dominant role in most patients. One such mechanism involves the overexpression of B7-H3, a member of the B7 family of immune checkpoint ligands. Studies have shown that B7-H3 is overexpressed in approximately 65% of SCLC cases. This protein has been shown to inhibit T-cell activation and proliferation, contributing to the ability of tumors to evade immune surveillance [142].

In addition to B7-H3, other mechanisms of immune evasion in SCLC are linked to the YAP1 signaling pathway. Research has demonstrated that SCLC-Y, a subtype of SCLC, has the worst clinical outcomes, suggesting that YAP1 plays a crucial role in mediating immune escape in this subgroup. YAP1 promotes the expression of PD-L1, which leads to a cascade of immunosuppressive effects. Additionally, YAP1 impairs the cytotoxic activity and activation of immune cells and may even promote immune cell apoptosis, rendering them ineffective at recognizing and attacking tumor cells [143].

Another immune evasion mechanism in SCLC involves the downregulation of MHC-I expression on tumor cells. Typically, MHC-I molecules present tumor antigens to CTLs, triggering an immune response. However, the reduced expression of MHC-I in SCLC allows the tumor to evade detection by CTLs. Interestingly, this downregulation increases the sensitivity of SCLC to NK cells, which do not rely on MHC-I for recognition. However, SCLC tumors often lack NKG2D ligands (NKG2DLs) on their surface, preventing the activation of NK cells and allowing the tumor to escape immune surveillance. In this context, the use of histone deacetylation inhibitors has been explored as a therapeutic strategy. These inhibitors can induce the expression of NKG2DLs, thus promoting the activation and recruitment of NK cells to target tumors [144].

### Biomarkers

Given the aggressive nature and rapid progression of SCLC, there is a critical need for reliable biomarkers to monitor treatment efficacy in real time. Such markers would enable clinicians to assess therapeutic response regularly and guide timely adjustments to the treatment regimen, thereby optimizing patient outcomes. While PD-L1 expression is widely recognized as a predictor of the immunotherapy response in many cancers, it is expressed at lower levels in SCLC patients and has not been identified as a crucial factor in the SCLC immunoptherapy response [145].

The tumor mutation burden (TMB) is a known biomarker for predicting immunotherapy outcomes in various cancers. However, its predictive value in SCLC is still debated. Both the IMpower133 study [44] and the CASPIAN trial [45, 46] indicated that TMB cannot predict the efficacy of immunotherapy in ES-SCLC patients. In contrast, the CheckMate032 study suggested that patients with high TMB may benefit more from immunotherapy, highlighting the need for further research on its predictive potential in SCLC (Supplementary Fig. 1–2) [146, 147].

The expression of human leukocyte antigen (HLA)-II molecules on TILs is associated with longer recurrence-free survival (RFS) and a lower risk of lymph node metastasis [148]. However, no significant difference in RFS was observed between SCLC patients expressing HLA-Ⅱ and those not expressing HLA-II, suggesting that HLA-II may not be a strong predictor of immunotherapy response in SCLC patients [148].

The serum lactate dehydrogenase (LDH) level has emerged as a potential biomarker for predicting the efficacy of ICIs. In SCLC, patients with LDH levels below the upper limit of normal (ULN) demonstrated a significant clinical benefit from ICIs (HR = 0.79, 95% CI 0.69–0.89, *P* < 0.001) [149].

CD38 is a transmembrane glycoprotein expressed in both hematopoietic and nonhematopoietic cells. In SCLC patients, increased levels of CD38 are correlated with increased expression of immunosuppressive markers such as FOXP3, PD-1, and CTLA-4. These findings suggest that CD38 may serve as a biomarker for predicting the response to ICIs in SCLC patients [150]. High CD38 may be a marker of patients who are less likely to respond to ICIs, although further validation is needed.

The number and characteristics of circulating tumor cells **(**CTCs) in the blood may provide real-time feedback on treatment efficacy. One study revealed that after one cycle of chemotherapy, the number of Bcl-2^+^/CD45^−^ CTCs significantly decreased in SCLC patients but increased as the disease progressed. Similar dynamic changes during immunotherapy may aid in response monitoring before obvious clinical progression [151].

Genetic alterations affecting DNA repair, including mutations and deletions related to DDR pathways, have been shown to correlate with a high TMB. These alterations may serve as predictive markers for ICIs response in SCLC, further emphasizing the role of DNA repair mechanisms in modulating immunotherapy efficacy [152].

N6-methyladenosine (m6A) is an epigenetic modification that has been linked to tumor progression and drug resistance. A retrospective study in other cancers suggested that m6A may be a valuable predictor of patient outcomes, although this topic has not been assessed in SCLC. Future research into m6A in SCLC may provide insights into its potential as a biomarker for immunotherapy response prediction and treatment selection [153].

The rapid progression of SCLC indicates the need for frequent monitoring of biomarkers to assess treatment efficacy and guide decisions about therapy adjustments. Currently, SLFN11, LDH, TMB, and PD-L1 expression are being used or investigated for their potential to predict treatment response. However, the predictive value of these biomarkers varies across clinical trials (Supplementary Fig. 1–2). In addition, emerging markers, such as CD38, DDR mutations, and m6A, may further refine patient management, especially as more research is conducted into their clinical applications. Given the convenience of routine blood tests, research groups have recently explored the relevance of the Lung Immune Prognostic Index (LIPI), which is calculated according to the derived neutrophil-to-lymphocyte ratio (dNLR) and LDH levels, to predict the immunotherapy response of SCLC patients [154]. The integration of multiple biomarkers into personalized treatment frameworks may pave the way for more precise and effective therapeutic regimens tailored to the molecular and immunological profiles of individual SCLC patients.

## Future directions and research needs

Despite therapeutic advances, including the use of ICIs, the PFS and OS of SCLC patients remain unsatisfactory, highlighting the urgent need for more effective and durable treatment strategies. Therapeutic resistance remains a major challenge in SCLC management, further limiting the efficacy of current interventions [138]. Additionally, the TIME in SCLC remains poorly characterized, posing significant barriers to the optimization of immunotherapeutic approaches. Given these challenges, the development of novel therapeutic strategies is crucial for improving survival outcomes and enhancing the quality of life of patients with SCLC.

One area of research focuses on the phenomenon of histological transformation—when an NSCLC changes into SCLC during treatment. This phenomenon is most commonly observed in some epidermal growth factor receptor (EGFR)-mutated lung adenocarcinoma patients who develop resistance to EGFR inhibitors, after which their tumors transform into SCLC. This process is often preceded by *RB1* and *TP53* mutations, with earlier events involving PIK3CA mutations and upregulation of the PI3K signaling pathway [155, 156]. Notably, such transformation can occur without new DNA mutations. Instead, transcriptional reprogramming, including the upregulation of PRC2 complex activity and genes associated with the PI3K/AKT and NOTCH pathways, plays a pivotal role. Furthermore, SCLC transformation may arise through an EMT stem-like state [157]. Notably, in *TP53-* and *RB1*-inactivated lung cancer cell lines, inhibition of exportin 1 (XPO1) has been shown to prevent SCLC transformation and increase the efficacy of chemotherapy [158, 159].Additionally, histological transitions from other lung cancer types to SCLC may also be mediated by noncanonical integrin signaling through ITGB2 [160]. An improved understanding of these transformation processes and related intervention strategies may prevent some cases of acquired resistance and is a key research area.

Another major effort involves the refinement of the molecular classification of SCLC. Traditional SCLC is currently subdivided into four molecular subtypes on the basis of transcription factor expression: ASCL1 (SCLC-A), NEUROD1 (SCLC-N), POU2F3 (SCLC-P), and YAP1 (SCLC-Y) [161, 162]. Each subtype appears to have unique vulnerabilities. For example, SCLC-P cells are sensitive to PARP inhibitors, whereas SCLC-I cells are more responsive to ICIs [163]. Furthermore, DLL3, a transcriptional target of ASCL1, represents a promising therapeutic target for SCLC-A [164]. As noted previously aSCLC, which displays widespread chromothripsis, appears particularly susceptible to temozolomide therapy, reflecting the genomic fragility of this subtype [67]. Dynamic monitoring of SCLC molecular phenotypes is critical for tailoring treatment strategies. Inhibition of the mSW/SNF complex, which specifically targets SMARCA4/2 ATPases and BRD4, has been shown to impede SCLC-P tumor growth. Similarly, targeting the POU2AF2-SWI/SNF axis, a key driver of SCLC-P, has emerged as a potential therapeutic strategy, with EZH2 inhibitors demonstrating efficacy in disrupting this pathway [165, 166]. Importantly, SCLC subtypes exhibit plasticity over time; that is, tumors can shift from one phenotype to another over time or under therapeutic pressure. MYC overexpression, for example, can induce transitions from SCLC-A to SCLC-N and subsequently to SCLC-Y, and the loss of KDM6A promotes a shift from the ASCL1 type to the NEUROD1 type [167, 168]. Such shifts can alter therapeutic efficacy. This dynamic behavior means that future SCLC treatments may need to be adaptive, that is, repeated tumor profiling may be needed to capture changes in tumor type.

Finally, researchers are turning to advanced technologies to better characterize SCLC and discover new targets. Multiomics approaches, including proteomics, provide insights beyond what DNA and RNA analyses can offer. Proteomic analyses have identified HMGB3 as a potential prognostic biomarker, with elevated expression linked to DNA repair and tumor progression. A recent classification based on the fold change (FC) further stratified SCLC into S-I, S-II, and S-III subtypes, revealing distinct chemotherapy sensitivities—with S-I being most sensitive and S-III being most resistant [169, 170]. As these tools are refined, they may enable further personalization of treatment according to the evolving molecular profile of each patient’s tumor.

## Conclusion

SCLC remains a highly aggressive malignancy with limited effective treatments and a high recurrence rate despite standard platinum-based chemotherapy. While ICIs have revolutionized cancer treatment, their efficacy in SCLC is restricted to a subset of patients, highlighting the need for novel therapeutic strategies. Recent advances in multiomics and spatial transcriptomics have refined our understanding of SCLC heterogeneity, uncovering new therapeutic targets and improving patient stratification. Emerging biomarkers, such as cell-free DNA (cfDNA) methylation patterns, offer a minimally invasive approach for monitoring disease progression and treatment response, thereby facilitating the development of personalized interventions [171–173]. Combination strategies integrating immunotherapy with chemotherapy, radiotherapy, and targeted agents shows promise. However, challenges remain in optimizing safety, improving efficacy, and overcoming resistance.

Despite these challenges, recent progress in SCLC immunotherapy research provides unprecedented hope for patients. With continuous evolution of innovative technologies and strategies, the therapeutic landscape of SCLC is poised for transformation. Future advances in genomics, transcriptomics, and temporal dynamics analysis are expected to drive the development of precision medicine strategies, enabling treatments tailored to the unique molecular and temporal characteristics of each patient.

## Supplementary Information

Supplementary Material 1

## Data Availability

Not applicable.

## Abbreviations

SCLC: Small Cell Lung Cancer
LS-SCLC: Limited Stage-Small Cell Lung Cancer
ES-SCLC: Extensive-Stage Small Cell Lung Cancer
TIME: Tumor-Immune Microenvironment
ICIs: Immune Checkpoint Inhibitors
CTLA-4: Cytotoxic T-lymphocyte-Associated protein 4
PD-1/PD-L1: Programmed Cell Death Protein 1 or its Ligand
EP: Etoposide and Platinum-based chemotherapy
ECM: Extracellular Matrix
CTLs: Cytotoxic T lymphocytes
TCR: T Cell Receptor
MHC-I: Major Histocompatibility Complex Class I
LUAD: Lung Adenocarcinoma
LUSC: Lung Squamous Cell Carcinoma
VEGF: Vascular Endothelial Growth Factor
TAMs: Tumor-Associated Macrophages
IL-6: Interleukin-6
IL-10: Interleukin-10
STAT3: Signal Transducer and Activator of Transcription 3
EZH2: Enhancer of Zeste Homolog 2
H3K27: Histone H3 at Lysine 27
NK: Natural killer
TREM2: Triggering Receptor Expressed on Myeloid cells 2
Tregs: Regulatory T cells
MDSCs: Myeloid-Derived Suppressor Cells
ARG1: Arginase 1
ROS: Reactive Oxygen Species
NO: Nitric Oxide
LILRB2: Leukocyte Immunoglobulin-Like Receptor B2
FFAR2: Free Fatty Acid Receptor 2
ATRA: All-Trans Retinoic Acid
TILs: Tumor-Infiltrating Lymphocytes
PFS: Progression-Free Survival
HR: Hazard Ratio
OS: Overall Survival
CI: Confidence Interval
PCI: Prophylactic Cranial Irradiation
HA-PCI: Hippocampal Avoidance-Prophylactic Cranial Irradiation
LDCT: Low-Dose Computed Tomography
LDRT: Low-Dose Radiotherapy
DLL3: Delta-Like Ligand 3
aSCLC: atypical SCLC
ADC: Antibody-Drug Conjugates
BiTE: Bispecific T-cell Engagers
CAR-T: Chimeric Antigen Receptor T-cell
Rova-T: Rovalpitumab Tesirine
IL-18: Interleukin-18
PARP: Poly (ADP-ribose) Polymerase
TIGIT: T-cell Immunoglobulin and ITIM domain
LSD1: Lysine-Specific Demethylase 1
H3K4: Histone H3 at lysine 4
CDYL: Chromodomain Y-like
LAG-3: Lymphocyte-Activation Gene 3
HNSCC: Head and Neck Squamous Cell Carcinoma
DHX9: DExD/H-box helicase 9
BRD4: Bromodomain-Containing protein 4
BETis: Bromodomain and Extraterminal Domain Inhibitors
GD2: Disialoganglioside
iPSCs: Derived from Pluripotent Stem Cells
Trop-2: Trophoblast cell surface antigen 2
EMT: Epithelial-Mesenchymal Transition
CLDN: Claudin
EphA2: Erythropoietin-producing hepatocellular A2
EndMT: Endothelial-to-Mesenchymal Transition
BET: Bromodomain and Extra Terminal
CNTN-1: Contactin 1
SLFN11: Schlafen 11
ATR: Ataxia Telangiectasia mutated and Rad3-related
CTCs: Circulating Tumor Cells
CHK1: Checkpoint Kinase 1
DDR: DNA Damage Response
P-gp: P-glycoprotein
TMB: Tumor Mutation Burden
HLA: Human Leukocyte Antigen
LDH: Serum Lactate Dehydrogenase
ULN: Upper Limit of Normal
m6A: N6-methyladenosine
LIPI: Lung Immune Prognostic Index
dNLR: derived Neutrophil-to-Lymphocyte Ratio
EGFR: Epidermal Growth Factor Receptor
XPO1: Exportin 1
FC: Fold Change
ITH: Intratumor Heterogeneity
β2M: β2-microglobulin
TGF-β: Transforming Growth Factor beta
IFN-β: Interferon-beta
CXCL9: CXC Motif Chemokine Ligand 9
CXCL10: CXC Motif Chemokine Ligand 10
SIRPα: Signal Regulatory Protein Alpha
GZMB: Granzyme B
FASL: FAS Ligand
NCID: Notch Intracellular Domain
Co-A: Coactivator
MAML: Mastermind-like
CSL: CBF1/Suppressor of hairless/Lag1
Co-R: Corepressor
CAR-T: Chimeric Antigen Receptor T cell
AEs: Adverse Events
GGT: Gamma-Glutamyl Transpeptidase
NR: Not Reported
AST: Aspartate Aminotransferase
DLTs: Dose Limiting Toxicities
MTD: Maximum Tolerated Dose
bTMB: Peripheral blood Tumor Mutational Burden
tTMB: tissue Tumor Mutation Burden
ULN: Upper Limit of Normal
