# Supplementary material for: Immunotherapy for small cell lung cancer: current challenges and prospects
Source: Exp Hematol Oncol. 2025 Nov 5;14:130. doi: 10.1186/s40164-025-00720-w (PMC12590821; doi:10.1186/s40164-025-00720-w)
Supplement: Supplementary file 1 — Supplementary Material 1 [file 40164_2025_720_MOESM1_ESM.docx]

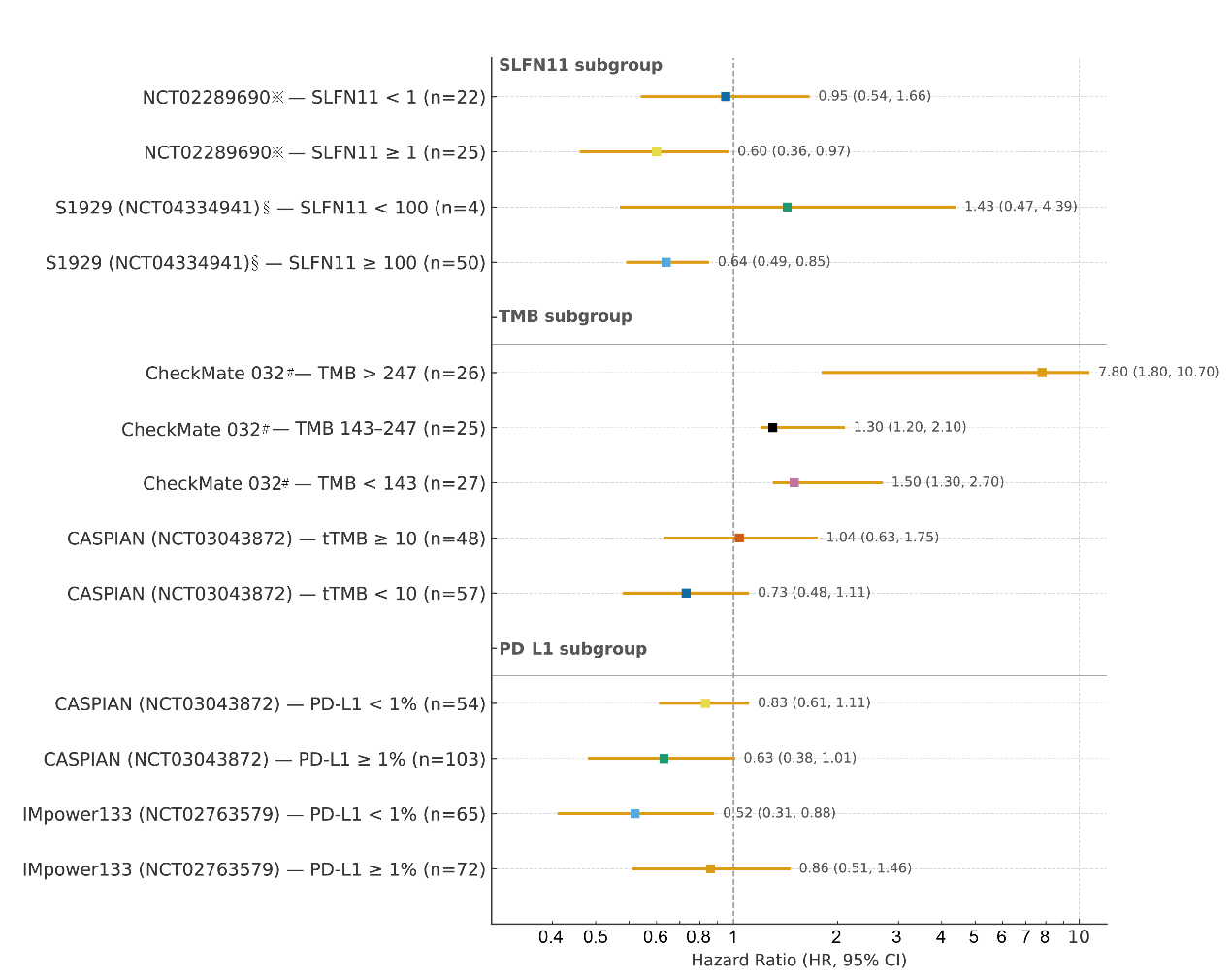


Supplementary Fig. 1 Subgroup analyses of PFS by SLFN11, TMB, and PD-L1 expression across different clinical trials.

※ NCT02289690 and § S1929 (NCT04334941): HRs are presented with 80%CI.

# CheckMate 032 reported the median PFS 95%CI.
Abbreviations: PFS, progression-free survival; OS, overall survival; bTMB, peripheral blood tumor mutational burden; tTMB, tissue tumor mutation burden; ULN, upper limit of normal; LDH, lactate dehydrogenase. HR, hazard ratio; CI: confidence interval.


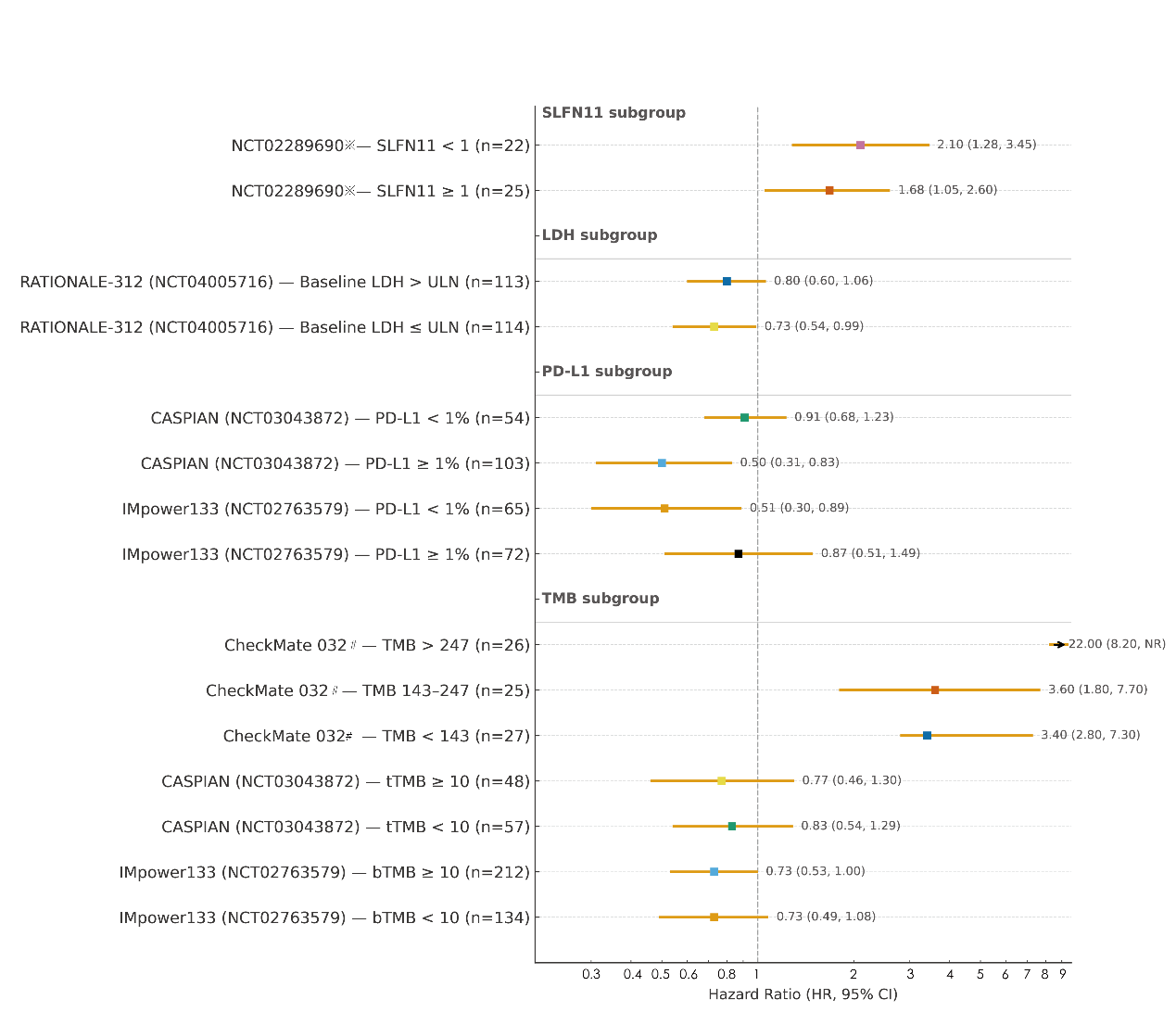


Supplementary Fig. 2 Subgroup analyses of OS by SLFN11, TMB, and PD-L1 expression across different clinical trials.

※ NCT02289690 and § S1929 (NCT04334941): HRs are presented with 80%CI.

# CheckMate 032 reported the median OS 95%CI.

Abbreviations: OS, overall survival.
